# Effect of recombinant IL-10 on cultured fetal rat alveolar type II cells exposed to 65%-hyperoxia

**DOI:** 10.1186/1465-9921-12-68

**Published:** 2011-05-24

**Authors:** Hyeon-Soo Lee, Chun-Ki Kim

**Affiliations:** 1 Department of Pediatrics, Kangwon National University Hospital, Kangwon National University School of Medicine, 17-1 Hyoja3-dong, Chuncheon, Kangwon 200-947, South Korea; 2 Institute of Medical Sciences, Kangwon National University School of Medicine, 17-1 Hyoja3-dong, Chuncheon, Kangwon 200-947, South Korea; 3 Medical and Bio-Materials Research Center, Kangwon National University School of Medicine, 192-1 Hyoja2-dong, Chuncheon, Kangwon 200-701, South Korea; 4 Department of Molecular and Cellular Biochemistry, Kangwon National University School of Medicine, 192-1 Hyoja2-dong, Chuncheon, Kangwon 200-701, South Korea

## Abstract

**Background:**

Hyperoxia plays an important role in the genesis of lung injury in preterm infants. Although alveolar type II cells are the main target of hyperoxic lung injury, the exact mechanisms whereby hyperoxia on fetal alveolar type II cells contributes to the genesis of lung injury are not fully defined, and there have been no specific measures for protection of fetal alveolar type II cells.

**Objective:**

The aim of this study was to investigate (a) cell death response and inflammatory response in fetal alveolar type II cells in the transitional period from canalicular to saccular stages during 65%-hyperoxia and (b) whether the injurious stimulus is promoted by creating an imbalance between pro- and anti-inflammatory cytokines and (c) whether treatment with an anti-inflammatory cytokine may be effective for protection of fetal alveolar type II cells from injury secondary to 65%-hyperoxia.

**Methods:**

Fetal alveolar type II cells were isolated on embryonic day 19 and exposed to 65%-oxygen for 24 h and 36 h. Cells in room air were used as controls. Cellular necrosis was assessed by lactate dehydrogenase-release and flow cytometry, and apoptosis was analyzed by TUNEL assay and flow cytometry, and cell proliferation was studied by BrdU incorporation. Release of cytokines including VEGF was analyzed by ELISA, and their gene expressions were investigated by qRT-PCR.

**Results:**

65%-hyperoxia increased cellular necrosis, whereas it decreased cell proliferation in a time-dependent manner compared to controls. 65%-hyperoxia stimulated IL-8-release in a time-dependent fashion, whereas the anti-inflammatory cytokine, IL-10, showed an opposite response. 65%-hyperoxia induced a significant decrease of VEGF-release compared to controls, and similar findings were observed on IL-8/IL-10/VEGF genes expression. Preincubation of recombinant IL-10 prior to 65%-hyperoxia decreased cellular necrosis and IL-8-release, and increased VEGF-release and cell proliferation significantly compared to hyperoxic cells without IL-10.

**Conclusions:**

The present study provides an experimental evidence that IL-10 may play a potential role in protection of fetal alveolar type II cells from injury induced by 65%-hyperoxia.

## Introduction

Administration of high concentrations of oxygen is a therapeutic mainstay for premature infants with respiratory distress syndrome since birth. However, prolonged exposure to hyperoxia, by generating excess reactive oxygen species, can generate lung injury [1-5] that leads to bronchopulmonary dysplasia (BPD) in preterm infants [6]. BPD has a multifactorial etiology, but one of the most immediate causes of BPD is lung injury imposed by hyperoxia [7], of which major biological effects include cell death and inflammatory response [8].

Alveolar type II cells are key components of alveolar structure. They participate in innate immune response by secreting chemokines and cytokines and are responsible for fluid homeostasis in alveolar lumen and restoration of normal alveolar epithelium after acute lung injury [9]. Hence, alveolar type II cells are the critical target of hyperoxia-mediated lung injury, and the rate of alveolar type II cell death is a critical factor determining the capacity of the epithelium to repair damage and should be related to the development of BPD [10]. Previous *in vitro *study of adult alveolar type II cells has demonstrated that 95%-hyperoxia increased lactate dehydrogenase (LDH)-release greatly compared to normoxic cells [11].

Hyperoxia-induced lung injury is characterized by lung edema, extensive inflammatory response and destruction of the alveolar-capillary barrier [5,12-14]. These effects are orchestrated by cytokines which amplify inflammatory cell influx into the lungs [15]. Increased level of pro-inflammatory cytokines and chemokines such as IL-8, TNFα, IL-1β, IL-6, IL-16, macrophage inflammatory protein (MIP-1) and monocyte chemoattractant protein (MCP-1) have been demonstrated in airway secretions of preterm infants with BPD [16]. IL-8, which is released by alveolar macrophages, fibroblasts, type II cells and endothelial cells, is considered as the most important chemotactic factor during the acute phase of lung inflammation [17,18]. In contrast, IL-10 is an anti-inflammatory cytokine that regulates the production of pro-inflammatory cytokines [9]. Recently, there have been growing concerns regarding the inability to regulate inflammation as a factor in development of BPD in preterm infants [19]. These concerns are based on previous evidence showing reduced response of IL-10 in bronchoalveolar lavage fluids of preterm infants with BPD [20,21].

In recent years, the features of BPD have changed. The lesions of altered patterns of atelectasis, overinflation and extensive fibroproliferation in "old" "BPD" have been replaced in "new" "BPD" with marked alveolar and capillary hypoplasia [22], resulting in developmental arrest of the lungs [23]. It is clear that coordination of distal lung vasculogenesis and alveolarization is essential for lung development [24], therefore, they are strongly considered to be under paracrine regulation, while VEGF expression reduced by hyperoxia is presumed to be mainly due to suppressed expression by alveolar type II cells [25].

We previously reported that recombinant IL-10 (rIL-10) administration is effective in attenuating type II cell injury induced by high amplitude stretch by reducing apoptosis and IL-8-release in fetal alveolar type II cells (FATIICs) [26]. Herein, we investigate cell death and inflammatory response in FATIICs exposed to sublethal hyperoxia, and further evaluate the effect of IL-10 administered to these exposed FATIICs, using an *in vitro *model in which rat FATIICs are isolated on embryonic day 19 (E19) of gestation (transition from canalicular to saccular stages of lung development).

## Methods

### Cell isolation, hyperoxia protocol and treatment procedure

Fetal rat lungs were obtained from time-pregnant Sprague-Dawley rats (Daehan Biolink, Eumsung, South Korea) on E19 (term = 22 days). Animal care and experimental procedures were performed in accordance with the Guidelines for Animal Experimentation of Kangwon National University School of Medicine with approval of the Institutional Animal Care. Extracted tissues were finely minced and digested with 0.5 mg/ml collagenase type I and 0.5 mg/ml collagenase type IA (Sigma Chemical Co., St. Louis, MO, USA) with vigorous pipetting for 15 min at 37°C. After collagenase digestion, cell suspensions were sequentially filtered through 100-, 30-, and 20-μm nylon meshes using screen cups (Sigma Chemical Co., St. Louis, MO, USA). The filtrate from 20-μm nylon mesh, containing mostly fibroblasts, was discarded. Clumped non-filtered cells from the 30- and 20-μm nylon meshes were collected after several washes with DMEM (Dulbecco's Modified Eagle Medium) to facilitate filtration of non-epithelial cells. Further type II cell purification was achieved by incubating cells in 75-cm^2 ^flasks for 30 min. Non-adherent cells were collected and cultured overnight in 75-cm^2 ^flasks containing serum-free DMEM. Purity of the type II cell fraction was determined to be 90 ± 5% by microscopic analysis of epithelial cell morphology and immune-blotting for cytokeratin/surfactant protein-C and vimentin as markers of epithelial cells and fibroblasts respectively [27]. After overnight culture, type II epithelial cells were harvested with 0.25%(wt/vol) trypsin in 0.4 mM EDTA and plated at a density of 10 × 10^5 ^cells/well on 6-well plates precoated with laminin [10 μg/ml]. Plates containing adherent cells were maintained for an additional 24 h in serum-free DMEM and then incubated in a culture chamber with ProOx Oxygen Controller with Low profile right angle sensor (BioSpherix, Redfiled, NY, USA). 65%-hyperoxia was applied for 24 h and 36 h, and cells grown in room air (5% CO_2_) were treated in an identical manner and served as controls. For the study of preincubation of rIL-10, the cells cultured in an identical manner were treated with rIL-10, at a concentration of 300 ng/ml for 1 h before hyperoxia exposure. The concentration of rIL-10, 300 ng/ml, was chosen based on our previous study showing that 300 ng/ml of rIL-10 affected greatly on reducing apoptosis and IL-8-release in FATIICs exposed to mechanical stretching [26]. And for the study to indentify the characteristics of the dual positive cells (Annexin V-positive and propidium iodide-positive) with FACscan, the cells cultured in an identical manner were incubated in 65%-hyperoxia and room air (5% CO_2_) at intervals of 6-12 h for 48 h.

### Lactate dehydrogenase assay

Lactate dehydrogenase (LDH) activity was measured using a CytoTox 96^® ^non-radioactive cytotoxicity assay (Promega, Madison, WI, USA), according to the manufacturer's protocol. This assay measures LDH-release into the supernatant upon cell lysis. The cytotoxicity was measured as % cytotoxicity [experimental LDH-release (OD490) per maximal LDH-release (OD490)]. LDH-releases were compared to the difference between the LDH-release in control samples. LDH was analyzed with a coupled enzymatic assay that results in the conversion of a tetrazolium salt into a red formazan product. The amount of color formed is proportional to the number of lysated cells. Absorbance at wavelength 490 nm was collected using a standard 96-well plate reader (Ultraspec 2100 pro, Amersham Pharmacia Biotech, Amersham, UK). LDH was quantified by dividing experimental LDH-release by maximal LDH-release (calculated after complete lysis of monolayers containing similar numbers of cells to the samples). This value was used as a common denominator for all samples tested.

### FACS analysis

FACS analysis was performed using an Annexin V-FITC apoptosis kit (BD Pharmingen, Franklin Lakes, NJ, USA), and analyzed by a flow cytometer (Becton Dickinson, Franklin Lakes, NJ, USA). FATIICs incubated at room air and 65%-hyperoxia in the presence and absence of 300 ng/ml of rIL-10 were washed, trypsinized and collected into each tube. Cells in trypsin were centrifuged at 1300 rpm for 3 min at 4°C, and resuspended in 1X Binding Buffer, and then 5 μl of FITC Annexin V (AV) and 5 μl of propidium iodide (PI) were added. After vortexing gently, the cells were incubated for 15 min at room air (25°C) in the dark. 400 ul of 1X binding buffer was added, and the cells were analyzed by flow cytometry.

### TUNEL assay

Detection and quantification of apoptotic cells were performed using terminal deoxynucleotidyl transferase-mediated dUTP-FITC nick-end labeling (TUNEL) by a fluorescein lable apoptosis detection system (Promega, Madison, WI, USA). Under experimental conditions, E19 monolayers were fixed in freshly prepared 4% paraformaldehyde in PBS for 25 min at 4°C, and permeabilized by immersion in 2.0% Triton X-100 in PBS. Positive controls were cells treated with Dnase I to induce DNA fragmentation. Monolayers were incubated at 37°C for 60 min in equilibration buffer, 2-deoxynucleotide 5'-triphosphate, and terminal deoxynucleotidyltransferase (TdT) enzyme as per manufacturer's protocol. A further control was prepared by omitting the TdT enzyme. Samples were washed in PBS, mounted with Vectashield mounting medium with PI (Vector Laboratories, Burlington, CA, USA), and analyzed by fluorescence microscopy. For quantification of apoptotic cells, 50 high-power fields per sample were analyzed. Areas from each membrane quadrant were randomly chosen and photographed. Cells containing green fluorescence and either nuclear condensation or chromatin fragmentation (without nuclear morphological changes) were identified as apoptotic cells. Results were expressed as TUNEL positive index (number of TUNEL positive cells per number of total cells).

### Western blot of caspase-3

E19 type II cells were exposed to 65%-hyperoxia for 24 h and 36 h, and cells in room air were used as controls. Monolayers were lysed with RIPA buffer containing protease inhibitors [28]. Lysates were centrifuged and total contents were determined by the bicinchoninic acid method. Equal amounts of protein lysate samples (20 μg) were fractionated by NU-PAGE Bis-Tris (4-12%) gel electrophoresis (Novex, SanDiego, CA, USA) and transferred to polyvinylidene difluoride membranes. Blots were hybridized with polyclonal antibody against the 11/17/20-kDa cleaved caspase-3 and 32-kDa full-length procaspase-3 (Santa Cruz Biotechnology, Santa Cruz, CA, USA) to detect activated caspase-3 and full-length caspase-3. Secondary antibody was conjugated with horseradish peroxidase, and blots were developed by exposing them to X-ray film. Membranes were then stripped and reprobed with actin antibody, and processed as described previously in this manuscript.

### Type II cell proliferation assay

Measurements of cell proliferation were analyzed by DNA incorporation of the thymidine analog 5-bromo'-deoxyuridine (BrdU) as described by the manufacturer (Boehringer Mannheim, Germany). Briefly, cultures (>90% confluence) were maintained in hyperoxic conditions or not, and immediately before each experiment, fresh medium containing 10 uM of BrdU labeling reagent was added to each well. At the end of each experiment, monolayers were washed with PBS and then fixed in 100% methanol for 20 min at -20°C. Cells were then washed and incubated with anti-BrdU antibody (negative controls were incubated with PBS) followed by incubation in fluorescein-conjugated secondary antibody and mounted with Vectashield mounting medium with DAPI (Vector Laboratories, Burlington, CA, USA). Slides were examined, photographed, and cells counted under Olympus bright-field fluorescence microscope. For quantification of BrdU-positive cells, 50 high-power fields per sample was analyzed.

### Concentration of cytokines and VEGF in supernatant

After experiments, cell culture medium was collected and stored at -80°C prior to analysis. Cytokine and VEGF concentrations in the supernatant were measured using commercial ELISA kits according to the manufacturer's recommendations (TNFα: Quantikine, R & D Systems, Minneapolis, MN, cat. # RTA00; IL-8<GRO/CINC-1>: Assay Designs, Ann Arbor, MI, cat. # 900-074; IL-10: Quantikine, R & D Systems, Minneapolis, MN, cat # R1000; VEGF: Quantikine, R & D systems, Minneapolis, MN, cat. # RRV00). Optical density was determined photometrically at 450 nm using the ELISA plate reader, EL_X_800 (Bio-Tek^® ^Instruments, Winooski, VT, USA). GRO/CINC-1 is a functional counterpart of human IL-8 from rat and structural and functional homology to human IL-8 [29]. ELISA kits had a minimum detectable concentration of 5 pg/ml for TNFα, 7.75 pg/ml for IL-8, 4.91 pg/ml for IL-10, and 8.4 pg/ml for VEGF. Cytokine levels were within the assay's detection limits in all samples.

### Real-time PCR (qRT-PCR)

Total RNA was extracted from E19 type II cells exposed to 65%-hyperoxia for 24 h and 36 h or parallel normoxic samples by a single-step method, and purified further with the Rneasy Mini Kit (Invitrogen, Carlsbad, CA, USA). Standard curves were generated for each primer set and housekeeping gene 18S ribosomal RNA. Linear regression revealed efficiencies between 96 and 99%. Therefore, fold expressions of hyperoxic samples relative to controls were calculated using the ΔΔC_T _method for relative quantification (RQ). Samples were normalized to the 18S rRNA. No differences in RQ values for 18S were found between control and hyperoxic samples. TaqMan primers were purchased from Assays-on-Demand™ Gene Expression Products (Applied Biosystems, Carlsbad, CA, USA). The following primers were used: TNFα (cat. #: Rn99999017_m1), GRO/CINC-1 (rat equivalent of IL-8) (5' primer:CATTAATATTTAACGATGTGGATGCG TTTCA;3'primer: GCCTACCATCTTTAAACTGCACAAT), IL-10 (cat. #: Rn 99999012_m1), VEGF (cat. #: Rn00582935_m1) and 18S (cat. #: Hs99999901_s1). Five micrograms of total RNA were reverse-transcribed into cDNA by the Superscript Double Stranded cDNA Synthesis kit (Invitrogen, Carlsbad, CA, USA). To amplify the cDNA by qRT-PCR, 5 μl of the resulting cDNA were added to a mixture of 25 μL of TaqMan Universal PCR Master Mix (Applied Biosystems, Carlsbad, CA, USA) and 2.5 μl of 20 × Assays-on-Demand™ Gene Expression Assay Mix containing forward and reverse primers and TaqMan-labeled probe (Applied Biosystems, Carlsbad, CA, USA). Reactions were performed in an ABI Prism 7000 Sequence Detection System (Applied Biosystems, Carlsbad, CA, USA). All assays were performed in duplicate.

### Statistical analysis

Results are expressed as mean ± SD from at least three experiments, using different litters for each experiment. For intergroup comparisons, data were analyzed with unpaired Student's *t*-test. A *p*-value < 0.05 was considered to be statistically significant.

## Results

### Effect of 65%-hyperoxia on fetal type II cell necrosis

**Figure 1:**
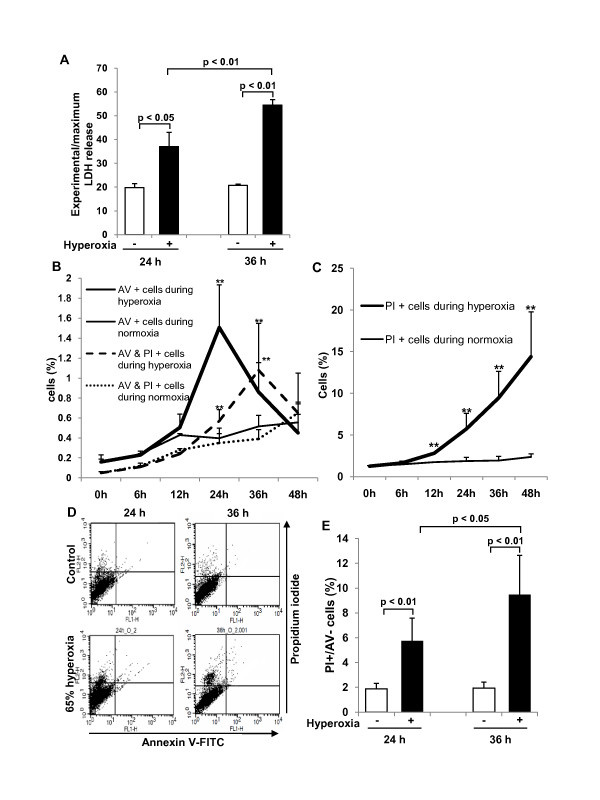
**Effect of 65%-hyperoxia on fetal type II cell necrosis**. **(A) **Graphical depiction showing LDH-release expressed as experimental minus background LDH-release divided by maximum LDH-release in hyperoxic and normoxic cells. The results are represented as the mean ± SD from 3 different experiments. **(B)** Graphical depiction showing the changes in the selective AV-positive cells and the dual positive (AV-positive and PI-positive) cells during normoxia and 65%-hyperoxia for 48 h. The results are represented as the mean ± SD from 3 different experiments. **; *p *< 0.01. **(C)** Graphical depiction showing the changes in the selective PI-positive cells during normoxia and 65%-hyperoxia for 48 h. The results are represented as the mean ± SD from 3 different experiments. **; *p *< 0.01. **(D) **Graphical depiction showing the distribution of necrotic cells (PI-positive and AV-negative) under normoxic and hyperoxic conditions. **(E) **Graphical depiction showing cellular necrosis (PI-positive and AV-negative) as a percentage of the total cell number in normoxic and hyperoxic cells. The results are represented as the mean ± SD from 6 different experiments.

Cell lysis analyzed by LDH-release into the supernatant significantly increased 1.9-fold after 24 h of hyperoxia (control = 19.8 ± 1.6 vs. hyperoxia = 37.0 ± 6.0; *p *< 0.05) and 2.6-fold after 36 h of hyperoxia (control = 20.7 ± 0.5 vs. hyperoxia = 54.5 ± 2.3; *p *< 0.01) when compared to controls (Figure 1A). We analyzed the characteristic distribution of FATIICs at intervals of 6-12 h during 65%-hyperoxia for 48 h with FACscan to identify the characteristics of the double stained [AV-positive and PI-positive] cells. As shown in Figure 1B, the double stained cells increased gradually during 65%-hyperoxia and peaked out at 36 h of hyperoxia, which were significantly greater compared to the normoxic cells (control = 0.39 ± 0.09 vs. hyperoxia = 1.08 ± 0.47; *p *< 0.05) and then decreased rapidly (Figure 1B). However, the dual positive cells in normoxic cells increased persistently after 36 h (Figure 1B). As shown in Figure 1B, selective AV-positive cells increased gradually during 65%-hyperoxia and peaked out at 24 h of hyperoxia, which were significantly higher compared to the normoxic cells (control = 0.40 ± 0.10 vs. hyperoxia = 1.51 ± 0.43; *p *< 0.01) and then decreased rapidly (Figure 1B). In contrast, selective PI-positive cells increased significantly in a time-dependent manner during 65%-hyperoxia compared to the normoxic cells (Figure 1C). According to these observations, the delayed increase at 36 h in the double positive cells may support the notion that these cells might be late apoptotic or necrotic, as they arose after the peak of early apoptotic cells; however, the percentage of the double positive cells were less than 1.5% of the FATIICs exposed to 65%-hyperoxia. Based on these data, pure necrotic cells were assessed by selective PI-positive cells with FACscan [30]. As shown in Figure 1D, 65%-hyperoxia increased the modest increase in selective PI-stained cells after 24 h and 36 h of hyperoixa (Figure 1D), and the percentage of cellular necrosis, as measured by selective PI staining [30], increased significantly after 24 h and 36 h of hyperoxia when compared to the control cells (24 h-control = 1.87 ± 0.45 vs. 24 h-hyperoxia = 5.74 ± 1.85; *p *< 0.01; 36 h-control = 1.94 ± 0.48 vs. 36 h-hyperoxia = 9.47 ± 3.17; *p *< 0.01) (Figure 1E).

### Effect of 65%-hyperoxia on fetal type II cell apoptosis

**Figure 2:**
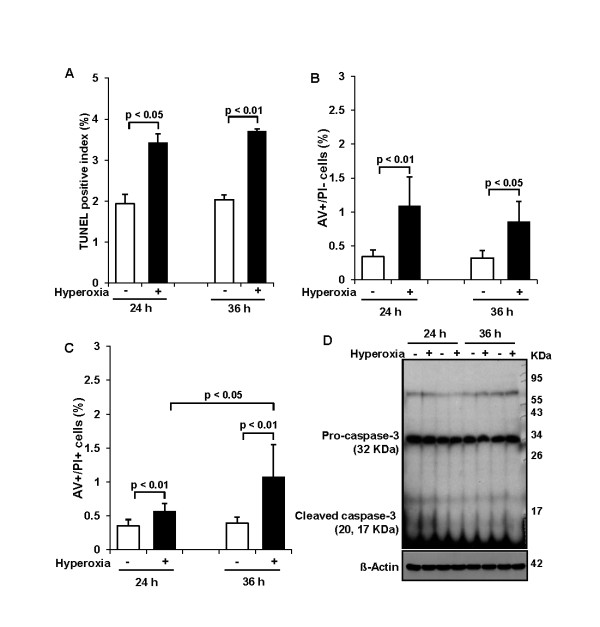
**Effect of 65%-hyperoxia on fetal type II cell apoptosis**. **(A) **Graphical depiction showing detection and quantification of DNA fragmentation analyzed by TUNEL assay in normoxic and hyperoxic cells. The results are represented as the mean ± SD from 3 different experiments. **(B) **Graphical depiction showing early apoptotic cells (selective AV-positive cells) as a percentage of the total cell number in normoxic and hyperoxic cells. The results are represented as the mean ± SD from 6 different experiments. **(C)** Graphical depiction showing late apoptotic or necrotic cells (AV-positive and PI-positive cells) as a percentage of the total cell number in normoxic and hyperoxic cells. The results are represented as the mean ± SD from 6 different experiments. **(D) **Western blot showing level of cleaved caspase-3 and abundance of full-length of procaspase-3 during 65%-hyperoxia.

DNA fragmentation assessed by TUNEL assay demonstrated that 65%-hyperoxia increased the apoptosis index 1.8-fold after 24 h (control = 1.9 ± 0.23 vs. hyperoxia = 3.4 ± 0.21; *p *< 0.05) and 1.9-fold after 36 h (control = 2.0 ± 0.12 vs. hyperoxia = 3.7 ± 0.06; *p *< 0.01) when compared to controls (Figure 2A). And the percentage of cells undergoing early apoptosis [selective AV-positive cells] assessed by FACscan had statistical increases in hyperoxic cells; however, the range was within 1.9% (24 h-control = 0.40 ± 0.10 vs. 24 h-hyperoxia = 1.51 ± 0.43; *p *< 0.01; 36 h-control = 0.52 ± 0.11 vs. 36 h-hyperoxia = 0.86 ± 0.29; *p *< 0.05) (Figure 2B). Similarly, the percentage of late apoptotic or necrotic cells (AV-positive and PI-positive cells) assessed by FACscan increased statistically in hyperoxic cells; however the range was within 1.5% (24 h-control = 0.35 ± 0.09 vs. 24 h-hyperoxia = 0.57 ± 0.11; *p *< 0.01; 36 h-control = 0.39 ± 0.09 vs. 36 h-hyperoxia = 1.08 ± 0.47; *p *< 0.01) (Figure 2C). In addition, western blots for caspase-3 showed that 65%-hyperoxia did not enhance level of cleaved caspase-3 and concomitantly did not decrease abundance of full-length procaspase-3 compared to control samples (Figure 2D).

### Effect of 65%-hyperoxia on fetal type II cell proliferation

**Figure 3:**
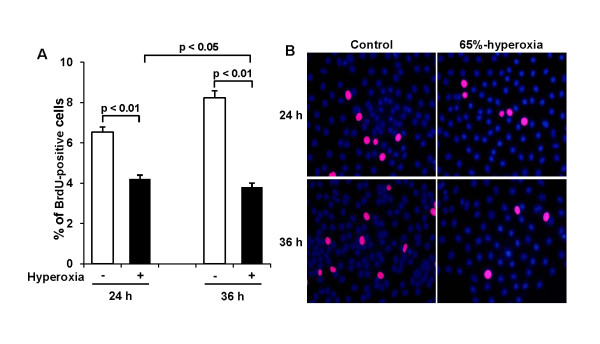
**Effect of 65%-hyperoxia on fetal type II cell proliferation**. **(A) **Graphical depiction showing BrdU-positive cells in hyperoxic and normoxic cells. The results are represented as the mean ± SD from 3 different experiments. (**B) **Representative fluorescence immunocytochemistry fields of E19 type II cells exposed to 65%-hyperoxia for 24 h and 36 h and parallel control samples. BrdU positive cells are labeled red. Nuclei were counterstained with DAPI (blue). Scale bar = 50 μm.

Cell proliferation was analyzed by DNA incorporation of the thymidine analog 5-bromo-2'-deoxyuridine (BrdU). 65%-hyperoxia decreased type II cell proliferation by 36% after 24 h (control = 6.5 ± 0.25 vs. hyperoxia = 4.2 ± 0.20; *p *< 0.01) and by 56% after 36 h (control = 8.2 ± 0.35 vs. hyperoxia = 3.8 ± 0.20; *p *< 0.01) when compared to controls (Figure 3A). Representative fluorescence immunocytochemistry fields from fetal lung type II cells exposed to 65%-hyperoxia for 24 h and 36 h and parallel normoxic cells are shown in Figure 3B.

### Effect of 65%-hyperoxia on VEGF and cytokine release from fetal type II cells

**Figure 4:**
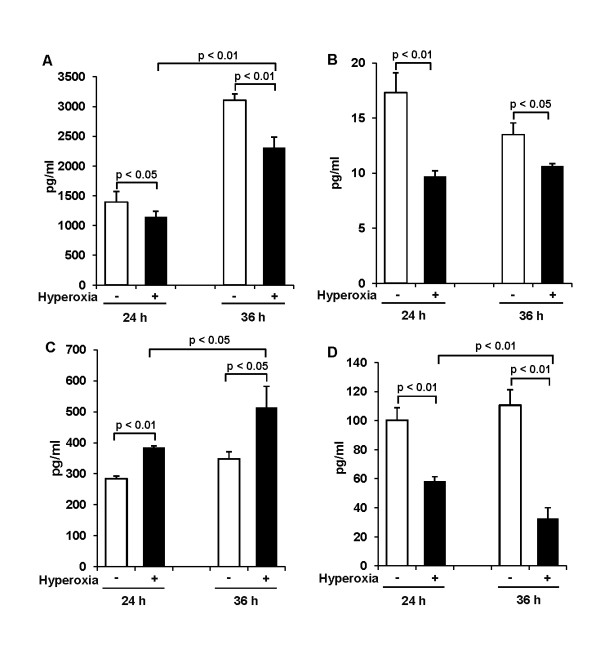
**Effect of 65%-hyperoxia on VEGF and cytokine release from fetal type II cells**. Supernatants were processed to assess VEGF **(A)**, TNFα **(B)**, IL-8 **(C)** and IL-10 **(D)** by ELISA in normoxic and hyperoxic cells. The results of IL-8, IL-10 and TNFα are represented as the mean ± SD from 3 different experiments, and the results of VEGF are represented as the mean ± SD from 6 different experiments.

VEGF and cytokines released into the supernatant were analyzed by ELISA. Results revealed that VEGF-release decreased significantly by 18% after 24 h (control = 1394.6 ± 175.9 vs. hyperoxia = 1143 ± 97.4; *p *< 0.05) and by 26% after 36 h of hyperoxia (control = 3105 ± 108.0 vs. hyperoxia = 2309 ± 178.1; *p *< 0.01) when compared to controls (Figure 4A). As shown in Figure 4B, TNFα levels were detected too low below 20 pg/ml in normoxic and hyperoxic conditions, and TNFα-release decreased significantly in hyperoxic samples compared to controls (24 h-control = 17.3 ± 1.80 vs. 24 h-hyperoxia = 9.7 ± 0.53; *p *< 0.01; 36 h-control = 13.5 ± 1.05 vs. 36 h-hyperoxia = 10.6 ± 0.22; *p *< 0.05) (Figure 4B). 65%-hyperoxia did not affect IL-1β or IL-6-release (data, not shown) from FATIICs. However, IL-8 increased 1.3-fold after 24 h (control = 284 ± 9.0 vs. hyperoxia = 385 ± 5.5; *p *< 0.01) and 1.5-fold after 36 h of hyperoxia (control = 348 ± 23.6 vs. hyperoxia = 513 ± 68.5; *p *< 0.05) when compared to controls (Figure 4C). In contrast, IL-10 decreased by 42% after 24 h (control = 100 ± 8.5 vs. hyperoxia = 58 ± 3.1; *p *< 0.01) and by 70% after 36 h of hyperoxia (control = 111 ± 10.5 vs. hyperoxia = 33 ± 7.4; *p *< 0.01) compared to controls (Figure 4D).

### Effect of 65%-hyperoxia on VEGF and cytokines gene expression

**Figure 5:**
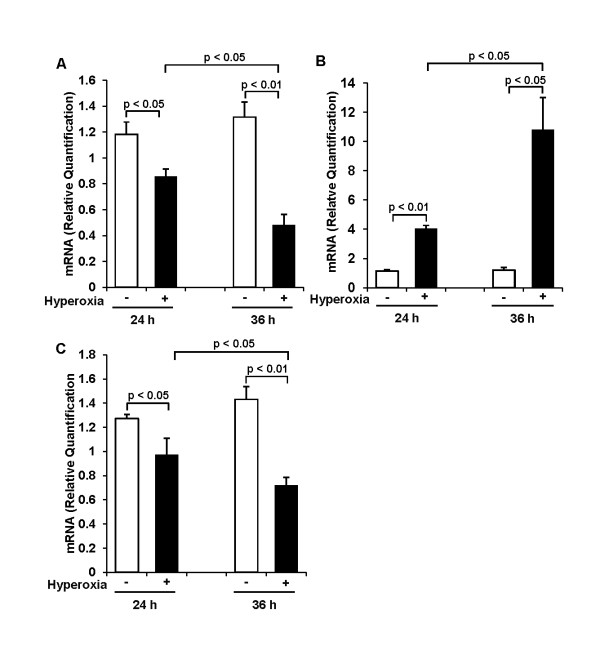
**Effect of 65%-hyperoxia on VEGF and cytokine genes expression**. Graphical depiction showing that 65%-hyperoxia upregulates VEGF **(A)** and IL-8 genes **(B)** and downregulates IL-10 **(C)** gene. The results of IL-8 and IL-10 are represented as the mean ± SD from 3 different experiments, and the results of VEGF are represented as the mean ± SD from 6 different experiments.

As a result of analyzing VEGF and cytokine genes expression using qRT-PCR, similar findings were observed with ELISA findings. As shown in Figure 5A, 65%-hyperoxia resulted in a significant decrease in VEGF mRNA by 18% and 64% after 24 h and 36 h, respectively when compared to controls (24 h-control = 1.18 ± 0.10 vs. 24 h-hyperoxia = 0.86 ± 0.06; *p *< 0.05; 36 h-control = 1.32 ± 0.12 vs. 36 h-hyperoxia = 0.48 ± 0.08; *p *< 0.01) (Figure 5A). And 65%-hyperoxia increased IL-8 mRNA 3.6-fold after 24 h (control = 1.13 ± 0.10 vs. hyperoxia = 4.03 ± 0.23; *p *< 0.01) and 9-fold after 36 h (control = 1.21 ± 0.18 vs. hyperoxia = 10.80 ± 2.21; *p *< 0.05) (Figure 5B), whereas it decreased IL-10 mRNA by 24% after 24 h (control = 1.27 ± 0.04 vs. hyperoxia = 0.97 ± 0.14; *p *< 0.05) and by 50% after 36 h (control = 1.43 ± 0.11 vs. hyperoxia = 0.72 ± 0.06; *p *< 0.01) (Figure 5C) when compared to controls.

### Effect of IL-10 preincubation of fetal type II cells before exposure to 65%-hyperoxia

**Figure 6:**
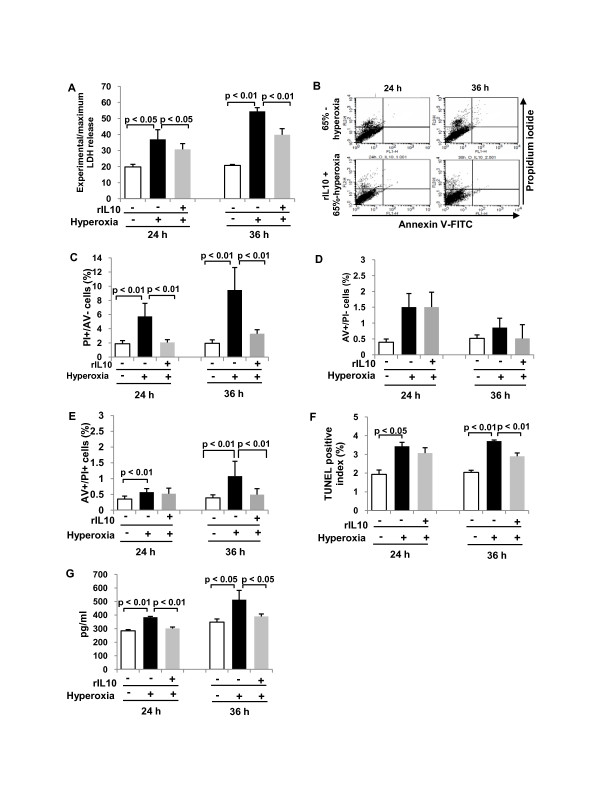
**IL-10 decreases cell death and IL-8 in fetal type II cells exposed to 65%-hyperoxia**. E19 cells were preincubated with a concentration of 300 ng/ml of rat rIL-10 before exposing to 65%-hyperoxia for 24 h and 36 h. Samples were processed to assess cellular necrosis, apoptosis and IL-8 released into the supernatant. **(A) **Graphical depiction showing LDH-release in treated and untreated cells. The results are represented as the mean ± SD from 3 different experiments. **(B) **Graphical depiction showing distribution of cellular necrosis measured by selective PI staining in treated and untreated cells. **(C) **Graphical depiction showing cellular necrosis (PI-positive and AV-negative cells) expressed as a percentage of the total cell number in treated and untreated cells. The results are represented as the mean ± SD from 6 different experiments. **(D) **Graphical depiction showing early apoptotic cells (selective AV-positive cells) assessed by FACscan in treated and untreated cells. The results are represented as the mean ± SD from 6 different experiments. **(E) **Graphical depiction showing late apoptotic or necrotic cells (AV-positive and PI-positive cells) assessed by FACscan in treated and untreated cells. The results are represented as the mean ± SD from 6 different experiments. **(F) **Graphical depiction showing DNA fragmentation assessed by TUNEL assay in treated and untreated cells. The results are represented as the mean ± SD from 3 different experiments. **(G) **Graphical depiction showing IL-8 released into the supernatant in treated and untreated cells. The results are represented as the mean ± SD from 3 different experiments.

**Figure 7:**
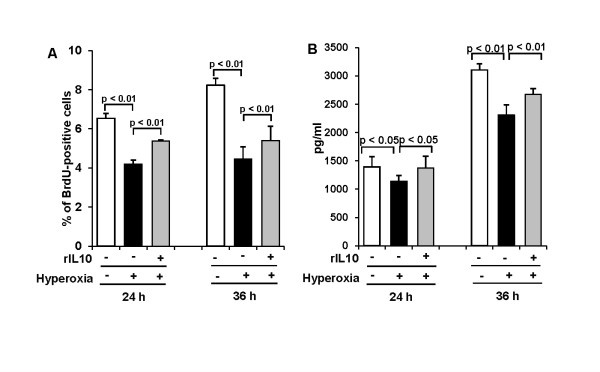
**IL-10 administration increases cell proliferation and VEGF-release in fetal type II cells exposed to 65%-hyperoxia**. E19 cells were preincubated with the concentration of 300 ng/ml of rat rIL-10 before exposing 65%-hyperoxia for 24 h and 36 h. Samples were processed to assess cell proliferation and VEGF released into the supernatant. **(A) **Graphical depiction showing the percentage of cells incorporating BrdU into nuclei in treated and untreated cells. The results are represented as the mean ± SD from 3 different experiments. **(B) **Graphical depiction showing VEGF released into the supernatant in treated and untreated cells. The results are represented as the mean ± SD from 6 different experiments.

According to the former data showing that 65%-hyperoxia induces increased cell death and decreased VEGF-release and cell proliferation and generates an imbalance between the pro-inflammatory cytokine, IL-8 and the anti-inflammatory cytokine, IL-10, in FATIICs. We evaluated whether preincubation of rIL-10 prior to hyperoxia would attenuate fetal type II cell injury secondary to 65%-hyperoxia. E19 type II cells were preincubated with 300 ng/ml of rat rIL-10 for 1 h prior to 65%-hyperoxia: **1) IL-10 administration decreases cell necrosis and IL-8 release**. As shown in Figure 6A, preincubation of rIL-10 significantly reduced cellular necrosis (measured by LDH-release) by 17% after 24 h of hyperoxia (untreated = 37.0 ± 5.99 vs. treated = 30.8 ± 3.56; *p *< 0.05) and by 27% after 36 h of hyperoxia (untreated = 54.5 ± 2.30 vs. treated = 39.8 ± 3.84; *p *< 0.01) respectively, when compared to cells without rIL-10 (Figure 6A). FACS analysis findings were similar with LDH-release, and showed cellular necrosis [PI-positive and AV-negative] greatly decreased in treated cells when compared to untreated cells (Figure 6B), and the percentage of cellular necrosis [PI-positive and AV-negative] significantly decreased in rIL-10-treated cells by 66% after 24 h and 36 h of hyperoxia (24 h-untreated = 5.74 ± 1.85 vs. 24 h-treated = 2.06 ± 0.39; *p *< 0.01; 36 h-untreated = 9.47 ± 3.17 vs. 36 h-treated = 3.30 ± 0.56; *p *< 0.01) (Figure 6C) when compared to untreated cells. However, early apoptotic cells [AV-positive and PI-negative cells] measured by FACScan were not affected significantly by rIL-10 (24 h-untreated = 1.51 ± 0.43 vs. 24 h-treated = 1.51 ± 0.47; 36 h-untreated = 0.86 ± 0.29 vs. 36 h-treated = 0.52 ± 0.44) (Figure 6D). In addition, the dual positive cells (late apoptotic or necrotic cells) measured by FACscan was affected by rIL-10 only at 36 h of hyperoxia (36 h-untreated = 1.08 ± 0.47 vs. 36 h-treated = 0.49 ± 0.19; *p *< 0.01) (Figure 6E). Similarly, apoptosis index assessed by TUNEL assay significantly decreased by 22% after 36 h when compared to hyperoxic cells without rIL-10 (36 h-untreated = 3.7 ± 0.06 vs. 36 h-treated = 2.9 ± 0.17; *p *< 0.01) (Figure 6F). As shown in Figure 6G, IL-8-release significantly decreased by 22% and 24% after 24 h and 36 h of hyperoxia respectively, in treated cells compared to untreated cells (24 h-untreated = 385 ± 5.5 vs. 24 h-treated = 302 ± 10.4; *p *< 0.01; 36 h-untreated = 513 ± 68.5 vs. 36 h-treated = 390 ± 18.5; *p *< 0.05) (Figure 6G). ***2) IL-10 administration increases cell proliferation and VEGF-release***. As shown in Figure 7A, cell proliferation increased 1.3-fold and 1.2-fold after 24 h and 36 h of hyperoxia, respectively in treated cells compared to untreated cells (24 h-untreated = 4.2 ± 0.20 vs. 24 h-treated = 5.4 ± 0.06; *p *< 0.01; 36 h-untreated = 4.5 ± 0.61 vs. 36 h-treated = 5.4 ± 0.72; *p *< 0.01) (Figure 7A). Similarly, VEGF-release increased 1.2-fold after 24 h and 36 h of hyperoxia, respectively in treated cells compared to untreated cells (24 h-untreated = 1143 ± 97.4 vs. 24 h-treated = 1376 ± 206.6; *p *< 0.05; 36 h-untreated = 2309 ± 178.1 vs. 36 h-treated = 2672 ± 102.0; *p *< 0.01) (Figure 7B).

## Discussion

The main findings of the present study are that 65%-hyperoxia of cultured FATIICs increased cellular necrosis and IL-8 production, while decreased VEGF production, cell proliferation and IL-10 production. Interestingly, preincubation with rIL-10 before hyperoxia protected FATIICs from injury secondary to 65%-hyperoxia by decreasing cellular necrosis and IL-8 production and increasing VEGF production and cell proliferation.

In our investigations, we selected 65%-hyperoxia, based on previous observation showing that 65%-hyperoxia exposure to newborn mice caused impairment of lung architecture in adult mice [31]. Therefore, in the current study, we investigated whether 65%-hyperoxia induces any injurious effect to FATIICs that are key components of the alveolar structure.

The present study showed that 65%-hyperoxia significantly increased LDH-release when compared to control samples. Exposure of hyperoxia causes direct oxidative cell damage through increased production of reactive oxygen species (ROS) [32]. Hence, lung damage secondary to hyperoxia is considered to be the direct results of increased intracellular ROS, which is accompanied by a secondary inflammatory response of the lungs [33]. All these pathologic alterations converge toward a central event, alveolar cell death [32].

Apoptosis, in the range of 0-3%, is a physiological event during lung morphogenesis [34]. Our investigations demonstrated statistically significant increase of TUNEL-positive fetal type II cells during 65%-hyperoxia when compared to controls. However, the increased levels of apoptosis measured by TUNEL assay ranged between 3.4% and 3.7%, which were within the normal physiological range. Similarly, early apoptotic cells measured by selective AV-positive staining [AV-positive and PI-negative] ranged between only 1.0% and 1.9% during 65%-hyperoxia. Our findings are somewhat similar to previous investigation demonstrating 2.5% of apoptotic cells in human fetal epithelial cells exposed to 95%-hyperoxia, that revealed decreased compared to normoxic cells [35].

However, there are some discrepancies between our results and others' regarding apoptotic activity in fetal alveolar epithelial cells exposed to hyperoxia. Haaften's group demonstrated high apoptotic index assessed by TUNEL assay, approximately 70%, in cultured rat type II cells at E19.5 after exposure to 85%-hyperoxia for 36 h [36], and Huang's group demonstrated 60% and 85% of apoptotic index in cultured rat type II cells at E21 after 95%-hyperoxia for 24 h and 48 h, respectively [37]. These discrepancies may be due to different doses of oxygen exposure and different experimental conditions. Apoptotic activities show oxygen dose dependence proportional to the amount of oxidants generated. Previously published data have shown lower concentrations of H_2_O_2 _do not induce TUNEL-positive apoptosis, only cell swelling, while intermediate doses of oxidants induce mixed modes of cell death, some dying by apoptosis, some by cell swelling [38-40]. Currently, it has not been clearly defined whether hyperoxia induces primarily apoptotic or non-apoptotic death *in vivo *and *in vitro*. However, previous reports have demonstrated that epithelial cells generally show aspects of necrosis during hyperoxia [1. 33, [41-45]]. These previous studies support our results in terms of fetal type II cell death including necrosis and apoptosis analyzed by LDH-release, TUNEL-assay and FACS analysis. In addition, different experimental conditions, such as cell culture plates used and the confluency of cells cultured may partially explain some differences between our apoptotic cells and those observed in other studies.

The current FACS' findings regarding the characteristics of FATIICs observed at intervals of 6-12 h during 65%-hyperoxia for 48 h showed that early apoptotic cells declined remarkably after 24 h of hyperoxia, whereas necrotic cells increased progressively in a time-dependent manner, which implies apoptotic activities may be suppressed as time progresses during the acute stage of sublethal hyperoxia while necrotic features are accelerated. Considering our observations with respect to fetal type II cell death, it may be speculated that cellular necrosis may be more relevant than apoptotic cell death in FATIICs during the initial stage of 65%-hyperoxia.

In addition, our investigations demonstrated that cleaved caspase-3 was not increased in FATIICs exposed to 65%-hyperoxia compared to controls, which is a similar finding to previous investigation of human fetal lung epithelial cells exposed to 95%-hyperoxia [35]. Current observation regarding caspase-3 may suggest that, during 65%-hyperoxia, caspases could prevent their own activation resulting in apoptosis in FATIICs so it could be possible that 65%-hyperoxia may trigger a caspase-independent cell death pathway [46]. This speculation is supported by our previous investigation showing that non-caspase proteolytic enzyme is greatly enhanced proportionally to the increase in fetal type II cell death during hyperoxia [47].

In addition to increased cell death, FATIICs cultured in 65%-oxygen demonstrated a decrease in cellular proliferation. This result is congruent with others that showed decreased cellular proliferation in FATIICs or newborn lung epithelial cells exposed to hyperoxia [37,48,49]. Current data on inhibition of type II cell proliferation in 65%-hyperoxia might be explained by DNA damage or cellular growth arrest [8]. Additionally, it is notable that failure of type II cells to proliferate during the 1st week of life may permanently alter postnatal lung growth during a critical period of postnatal lung development, which may play an important role in the evolution of BPD in preterm infants [50].

Our present data on VEGF expression in FATIICs showed significant decreases during 65%-hyperoxia for 36 h, which are consistent with data from premature lung explants [35,51]. Epithelial-mesenchymal interactions are considered to be necessary for lung vascular development [52], and alveolar type II cells undergo growth and differentiation in the presence of VEGF [53]. Therefore, expression of VEGF from alveolar epithelial cells is presumed to be responsible for alveolar development [54]. In the current study, hyperoxia was found to be capable of destroying FATIICs at the early stage of hyperoxia, which may contribute to the genesis of lung injury in preterm lungs. Although VEGF is known to act as a prototypic growth factor for endothelial and epithelial cells [53], growth factors may have the dual effect of either being protective or harmful; therefore, the growth factor including VEGF may be harmful if overexpressed. This lends support to the finding that VEGF decreases in bronchoalveolar lavage fluid (BALF) until 3 days from birth in premature newborns with respiratory distress syndrome, whereas it increases greatly (2.2-fold) in BALF of 1-week-old preterm newborns who had BPD compared to the levels observed at 3 days of life. In contrast, in premature newborns without BPD, VEGF at 1 week of age does not increase greatly (1.1-fold) compared to the levels observed at 3 days of age [55]. This increase in VEGF in preterm newborns with BPD is presumed to be caused by the increased production of VEGF possibly due to inflammatory cells, which may be the source of VEGF. Moreover, the excessive VEGF can act as a potent permeability factor. In addition, the location of the VEGF expression appears to be important according to previous observations which showed that increases in plasma VEGF were related to higher mortality [56], while increases in the levels of VEGF in the epithelial lining fluid were associated with the recovery of patients with acute respiratory distress syndrome [57]. Based on the previously reported evidence, the increase in VEGF in FATIICs preincubated with rIL-10 can be assumed to contribute to the protection of type II cells. This is also supported by previously published reports which demonstrated that VEGF production has a protective effect against hyperoxic acute lung injury [58].

Our investigation of cytokines showed very low levels of TNFα (< 20 pg/ml) in both normoxic and hyperoxic conditions, implying that the role of TNFα is not clear in hyperoxic injury, and cytokine release may not be TNFα-mediated in FATIICs during hyperoxia. Our speculation is supported by previous evidence showing that the blocking of TNFα function is not effective in reducing lung injury during hyperoxia [59]. In addition, our results of low levels of TNFα may explain partly why caspase 3 did not increase in FATIICs exposed to 65%-hyperoxia, based on previous evidence showing that inhibition of TNFα prevents hyperoxia-mediated activation of caspase 3 in type II cells [60]. However, hyperoxia affected IL-8 and IL-10-release in FATIICs. Our investigations showed that under normoxic conditions, IL-8 concentrations increased and IL-10 concentrations were nearly static overtime (see control samples at 24 h and 36 h in Figure 4C & Figure 4D); in contrast, under hyperoxic conditions, IL-8 increased significantly in a time-dependent manner, while IL-10 significantly decreased. Considering all of these responses, it may be suggested that under normoxic conditions, IL-10 may have a counterregulatory effect to inhibit inflammation, whereas under hyperoxic conditions, IL-10 failed to optimize its protective effect. Hence, it is more suspicious that balance between pro- and anti-inflammatory cytokines is shifted by a combination of an increase of pro-inflammatory cytokine and a stronger decrease of an anti-inflammatory cytokine rather than by increase in pro-inflammatory cytokines.

Our observations of IL-8 upregulation are consistent with previous observations showing that IL-8 was upregulated by oxygen tension in adult type II cells, which was independent of IL-1 receptor pathway [61], and IL-8 increased in adult human airway epithelial cells exposed to hyperoxia [62].

Our observation that preincubation with rIL-10 attenuated cellular necrosis and IL-8-release, and promoted cell proliferation and VEGF-release in FATIICs exposed to 65%-hyperoxia is both important and novel. Mechanisms by which IL-10 reduces type II cell injury secondary to hyperoxia are not clearly defined yet. However, some plausible explanations can be considered. First, IL-10 could directly affect IL-8 production by inhibiting the activation of NF-_k_B, a pivotal transcription factor modulating inflammation. Also the inhibitory effect of IL-10 on NF-_k_B could induce increases in cell proliferation by allowing entry to the S-1 phase. Second, IL-10 could indirectly influence IL-8 production through its effect on modulating IL-8 receptors. The effect of IL-10 on suppressing receptors of pro-inflammatory cytokines has been revealed in IL-10 knock-out mice [63]. Further, IL-8 receptors are involved in one of the pathways for genesis of intracellular oxygen radical species by binding to the tyrosine kinase receptors including PDGF (platelet-derived-growth-factor) and EGF (epidermal growth factor) located on the surface of type II cells [64]. Therefore, suppression of IL-8 receptors by IL-10 may contribute to the prevention of additional type II cell death. Third, the effect of IL-10 in reducing type II cell necrosis could be attributed to inhibition of proteolytic enzymes, which might contribute to increased VEGF production. Although our *in-vivo *experimental study suggests that IL-10 is a possible therapeutic option to target acute lung injury in preterm lungs, exploration of the optimal time and dose of IL-10 is required given on the previous observation that increased systemic inflammation and a worse outcome can result from higher doses of adenoviruses [65].

## Conclusions

This is the first experimental *in-vivo *study investigating two kinds of fetal type II cell injury, cell death and inflammatory response, which are both inducible during hyperoxia, and whether rIL-10 may be effective in attenuating two mechanisms of fetal type II cell injury secondary to hyperoxia.

In summary, our investigation suggests that 65%-hyperoxia of FATIICs induces increased cellular necrosis, decreased cell proliferation and VEGF production, and generates an imbalance between pro- and anti-inflammatory cytokines, and addition of rIL-10 reduces cellular necrosis and IL-8 production and promotes cell proliferation and VEGF production in FATIICs. Our *in vivo *studies imply that IL-10 might be a promising therapeutic agent for attenuating or even preventing acute lung injury secondary to hyperoxia in premature lungs.

## List of abbreviations

BPD: Bronchopulmonary dysplasia; LDH: Lactate dehydrogenase; rIL-10: recombinant interleukin-10; FATIICs: Fetal alveolar type II cells; E19: Embryonic day 19; FACS: Fluorescenc-activated cell sorting; AV: Annexin V; PI: Propidium iodide; TUNEL: Terminal deoxynucleotidyl transferase-mediated dUTP-FITC nick-end labeling; BrdU: 5-bromo'-deoxyuridine; VEGF: Vascular endothelial growth factor

## Competing interests

The authors declare that they have no competing interests.

## Authors' contributions

HSL and CKK did the animal studies. HSL has made substantial contributions to conception and design, acquisition of data, analysis, interpretation of data and manuscript development. HSL and CKK read and approved the final manuscript.

## References

[B1] Barazzone C Horowitz S Donati YR Rodriguez I Piguet PF Oxygen toxicity in mouse lung: pathways to cell death Am J Respir Cell Mol Biol 1998 19 573 581 9761753 10.1165/ajrcmb.19.4.3173

[B2] Barazzone C White CW Mechanism of cell injury and death in hyperoxia: role of cytokines and Bcl-2 family proteins Am J Respir Cell Mol Biol 2000 22 535 542 10783120 10.1165/ajrcmb.22.5.f180

[B3] O'Reilly MA Staversky RJ Huyck HL Watkins RH LoMonaco MB D'Angio CT Baggs RB Maniscalco WM Pryhuber GS Bcl-2 family gene expression during severe hyperoxia induced lung injury Lab Invest 2000 80 1845 1854 10.1038/labinvest.3780195 11140697

[B4] Ward NS Waxman AB Homer RJ Mantell LL Einarsson O Du Y Elias JA Interleukin-6 induced protection in hyperoxic acute lung injury Am J Respir Cell Mol Biol 2000 22 535 542 10783124 10.1165/ajrcmb.22.5.3808

[B5] Waxman AB Einarsson O Seres T Knickelbein RG Warshaw JB Johnston R Homer RJ Elias JA Targeted lung expression of interleukin-11 enhances murine tolerance of 100% oxygen and diminishes hyperoxia-induced DNA fragmentation J Clin Invest 1998 101 1970 1982 10.1172/JCI1337 9576762 PMC508784

[B6] Rush MG Hazinski TA Current therapy of bronchopulmonary dysplasia Clin Perinatol 1992 19 563 590 1526072

[B7] Jobe AH Ikegami M Mechanisms initiating lung injury in the preterm Early Hum Dev 1998 53 81 94 10.1016/S0378-3782(98)00045-0 10193929

[B8] Lee PJ Choi AM Serial review: Role of reactive oxygen and nitrogen species (ROS/RNS) in lung injury and diseases Free Rad Biol Med 2003 35 341 350 10.1016/S0891-5849(03)00279-X 12899937

[B9] Opal SM DePalo VA Anti-inflammatory cytokines Chest 2000 117 1162 1172 10.1378/chest.117.4.1162 10767254

[B10] Martin TR Frevert CW Innate immunity in the lungs Am Thorac Soc 2005 1 214 220 10.1513/pats.200508-090JS PMC2713330 16322590

[B11] Rahman I Mulier B Gilmour PS Watchorn T Donaldson K Jeffery PK MacNee W Oxidant-mediated lung epithelial cell tolerance: the role of intracellular glutathione and NF_k_b Biochemi Pharmacol 2001 62 787 794 10.1016/S0006-2952(01)00702-X 11551525

[B12] Corne JG Chupp CG Lee RJ Homer Z Zhu Q Chen B Ma Y Du F Roux J McArdle AB Waxman J Elias A IL-13 stimulates vascular endothelial cell growth factor and protects against hyperoxic acute lung injury J Clin Invest 2000 106 783 791 10.1172/JCI9674 10995789 PMC381393

[B13] Horowitz S Davis JM MacDonald JM, Dekker M From Lung injury when development is interrupted by premature birth Growth and Development 1997 NY: New York

[B14] Slutsky AS Lung injury caused by mechanical ventilation Chest 1999 116 9S 15S 10.1378/chest.116.suppl_1.9S 10424561

[B15] Bhandari V Elias JA Cytokines in tolerance to hyperoxia-induced injury in the developing and adult lung Free Radic Biol Med 2006 41 4 18 10.1016/j.freeradbiomed.2006.01.027 16781448

[B16] Speer CP Inflammation and bronchopulmonary dysplasia: a continuing story Semin Fetal Neonatal Med 2006 11 354 362 10.1016/j.siny.2006.03.004 16702036

[B17] Mourgeon E Isowa N Keshavjee S Zhang X Slutsky AS Liu M Mechanical stretch stimulates macrophage inflammatory protein-2 secretion from fetal rat lung cells Am J Physiol Lung Cell Mol Physiol 2000 279 L699 L706 11000130 10.1152/ajplung.2000.279.4.L699

[B18] Shanley TP Vasi N Denenberg A Regulation of chemokine expression by IL-10 in lung inflammation Cytokine 2000 12 1054 1064 10.1006/cyto.1999.0655 10880252

[B19] Schultz C Temming P Bucsky P Göpel W Strunk T Hätel C Immature anti-inflammatory response in neonates Clini Exp Immunol 2004 135 130 136 10.1111/j.1365-2249.2004.02313.x PMC1808915 14678274

[B20] Jones CA Cayabyab RG Kwong KY Stotts C Wong B Hamdan H Minoo P deLemos RA Undetectable interleukin (IL)-10 and persistent IL-8 expression early in hyaline membrane disease: a possible development basis for the predisposition to chronic lung inflammation in preterm newborns Pediatr Res 1996 39 966 975 10.1203/00006450-199606000-00007 8725256 PMC7101752

[B21] Kwong KY Jones CA Cayabyab R Lecart C Khuu N Rhandhawa I Hanley JM Ramanathan R deLemos RA Minoo P The effects of IL-10 on proinflammatory cytokine expression (IL-1beta and IL-8) in hyaline membrane disease (HMD) Clin Immunol Immunopathol 1998 88 105 113 10.1006/clin.1997.4510 9683557

[B22] Coalson JJ Pathology of new bronchopulmonary dysplasia Semin Neonatol 2003 8 73 81 10.1016/S1084-2756(02)00193-8 12667832

[B23] Coalson JJ Pathology of bronchopulmonary dysplasia Semin Perinatol 2006 30 179 184 10.1053/j.semperi.2006.05.004 16860157

[B24] Bourdon J Boucherat O Challey-Heu B Delacourt C Control mechanisms of lung alveolar development and their disorders in bronchopulmonary dysplasia Pediatr Res 2005 57 38 46 10.1203/01.PDR.0000159630.35883.BE 15817499

[B25] Maniscalo WM Watkins RH D'Angio CT Ryan RM Hyperoxic injury decreases alveolar epithelial cell expression of vascular endothelial growth factor (VEGF) in neonatal rabbit lung Am J Respir Cell Mol Biol 1997 16 557 567 9160838 10.1165/ajrcmb.16.5.9160838

[B26] Lee HS Wang Y Maciejewski BS Esho K Fulton C Sharma S Sanchez-Esteban J Interleukin-10 protects cultured fetal rat type II epithelial cells from injury induced by mechanical stretch Am J Physiol Lung Cell Mol Physiol 2008 294 L225 L232 18065656 10.1152/ajplung.00370.2007

[B27] Sanchez-Esteban J Wang Y Gruppuso PA Rubin LP Mechanical stretch induces fetal type II cell proliferation via an epidermal growth factor receptor-extracellular-regulated protein kinase signaling pathway Am J Respir Cell Mol Biol 2004 30 76 83 12829451 10.1165/rcmb.2003-0121OC

[B28] Sanchez-Esteban J Wang Y Filardo EJ Rubin LP Ingber DE Integrins β1, α6, and α3 contributes to mechanical strain-induced differentiation of fetal lung type II epithelial cells via distinct mechanisms Am J Physiol Lung Cell Mol Physiol 2006 290 343 350 10.1152/ajplung.00189.2005 16169900

[B29] Deng HS Mason N Auten R Jr Lung inflammation in hyperoxia can be prevented by antichemokine treatment in newborn rats Am J Respir Crit Care Med 2000 162 2316 2323 11112157 10.1164/ajrccm.162.6.9911020

[B30] Bhandari V Choo-Wing R Lee CG Zhu Z Nedrelow JH Chupp GL Zhang X Matthay MA Ware LB Homer RJ Lee PJ Geick A Fougerrolles AR Elias JA Hyperoxia causes angiopoietin 2-mediated acute lung injury and necrotic cell death Nat Med 2006 12 1286 1293 10.1038/nm1494 17086189 PMC2768268

[B31] Dauger S Ferkdadji L Saumon G Vardon G Peuchmaur M Gaultier C Gallego J Neonatal exposure to 65% oxygen durably impairs lung architecture and breathing pattern in adult mice Chest 2003 530 538 10.1378/chest.123.2.530 12576377

[B32] Pagano A Barazzone-Argiroff C Alveolar cell death in hyperoxia-induced lung injury Ann N Y Acad Sci 2003 1010 405 416 10.1196/annals.1299.074 15033761

[B33] Mantell LL Lee PJ Signal transduction pathways in hyperoixa-induced lung cell death Mol Genet Metab 2000 71 359 370 10.1006/mgme.2000.3046 11001828

[B34] Scavo LM Ertsey R Chapin CJ Allen L Kitterman JA Apoptosis in the development of rat and human fetal lungs Am J Respir Cell Mol Biol 1998 18 21 31 9448042 10.1165/ajrcmb.18.1.2744

[B35] Bustani P Hodge R Tellabati A Li J Pandya H Kotecha S Differential response of the epithelium and interstitium in developing human fetal lung explants to hyperoxia Pediatr Res 2006 59 383 388 10.1203/01.pdr.0000198774.79043.5c 16492976

[B36] Haaften T Byrne R Bonnet S Rochefort GY Akabutu J Bouchentouf M Rey-Parra GJ Haromy A Eaton F Chen M Hashimoto K Abley D Korbutt G Archer SL Thebaud B Airway delivery of mesenchymal stem cells prevents arrested alveolar growth in neonatal lung injury in rats Am J Respir Crit Care Med 2009 180 1131 1142 10.1164/rccm.200902-0179OC 19713449 PMC3269236

[B37] Huang B Fu H Yang M Fang F Kuang F Xu F Neuropeptide substance P attenuates hyperoxia-induced oxidative stress injury in type II alveolar epithelial cells via suppressing the activation of JNK pathway Lung 2009 187 421 426 10.1007/s00408-009-9177-z 19789913

[B38] Iwata M Myerson D Torok-Storb B Zager RA An evaluation of renal tubular DNA laddering in response to oxygen deprivation and oxidant injury J Am Soc Nephrol 1994 5 1307 1311 7893995 10.1681/ASN.V561307

[B39] Slater AFC Nobel CSI Orrenius S The role of intracellular oxidants in apoptosis Biochem Biophys Acta Mol Basis Dis 1994 1271 59 62 10.1016/0925-4439(95)00010-2 7599226

[B40] Ueda N Shah SV Endonuclease-induced DNA damage and cell death in oxidant injury to renal tubular epithelial cells J Clin Invest 1992 90 2593 2597 10.1172/JCI116154 1334979 PMC443419

[B41] Barazzone C Donati YR Rochat AF Vesin C Kan CD Pache JC Piguet PF Keratinocyte growth factor protects alveolar epithelium and endothelium from oxygen-induced injury in mice Am J Pathol 1999 154 1479 1487 10.1016/S0002-9440(10)65402-8 10329601 PMC1866589

[B42] Kanduc D Mittelman A Serpico R Sinigaglia E Sinha AA Natale C Santacroce R Di Corcia MG Lucchese A Dini L Pani P Santacroce S Simone S Bucci R Farber E Cell death: apoptosis versus necrosis (review) Intl J Oncol 2002 21 165 170 12063564

[B43] Kroemer G Dallaporta B Resche-Rigon M Mitochondrial death/life regulator in apoptosis and necrosis Annu Rev Physiol 1998 60 619 642 10.1146/annurev.physiol.60.1.619 9558479

[B44] Saraste A Pulkki K Morphologic and biological hallmarks of apoptosis Cardiovasc Res 2000 45 528 537 10.1016/S0008-6363(99)00384-3 10728374

[B45] Van Cruchten S Van Den Broeck W Morphologial and biochemical aspects of apoptosis, oncosis and apoptosis Am J Physiol 1999 276 G1 6 12196263 10.1046/j.1439-0264.2002.00398.x

[B46] Susin SA Daugas E Ravagnan L Samejima K Zamzami N Loeffler M Costantini P Ferri KF Irinopoulou T Prévost MC Brothers G Mak TW Penninger J Earnshaw WC Kroemer G Two distinct pathways leading to nuclear apoptosis J Exp Med 2000 192 571 580 10.1084/jem.192.4.571 10952727 PMC2193229

[B47] Lee HS Kim CK Cathepsin-B is activated as an executive protease in fetal alveolar type II epithelial cells exposed to hyperoxia Exp Mol Med 2011 43 223 229 10.3858/emm.2011.43.4.027 21415591 PMC3085741

[B48] Alphonse RS Vadivel A Coltan L Eaton F Barr AJ Dyck JRB Thebaud B Activation of Akt protects alveoli from neonatal oxygen-induced lung injury Am J Respir Cell Mol Biol 2011 44 146 154 10.1165/rcmb.2009-0182OC 20348209

[B49] Esquibies AE Bazzy-Asaad A Ghassemi F Nishio H Karihaloo A Cantley LG VEGF attenuates hyperoxic injury through decreased apoptosis in explanted rat embryonic lung Pediatr Res 2008 63 20 25 10.1203/PDR.0b013e31815b4857 18043510

[B50] Yee M Vitello PF Roper JM Staversky R Wright TW McGrath-Morrow SA Maniscalo WM Finkelstein JN O'Reilly MA Type II epithelial cells are critical target for hyperoxia-mediated impairment of postnatal lung developmemt Am J Physiol Lung Cell Mol Physiol 2006 291 L1101 1111 10.1152/ajplung.00126.2006 16861382

[B51] Muniscalo WM Watkins RH D'Angio CT Ryan RM Hyperoxic lung injury decreases alveolar epithelial cell exposure of vascular endothelial growth factor (VEGF) in neonatal lung Am J Respir Cell Mol Biol 1997 16 557 567 9160838 10.1165/ajrcmb.16.5.9160838

[B52] Gebb SA Shannon JM Tissue interactions mediate early events in pulmonary vasculogenesis Dev Dyn 2000 217 159 169 10.1002/(SICI)1097-0177(200002)217:2<159::AID-DVDY3>3.0.CO;2-9 10706140

[B53] Voelkel NF William V R Rubin MT Vascular endothelial growth factor in the lung Am J Physiol Lung Cell Mol Physiol 2006 290 209 221 10.1152/ajplung.00185.2005 16403941

[B54] Maniscalco WM Differential expression of VEGF mRNA splice variants in newborn and adult hyperoxic lung injury Am J Physiol Lung cell Mol Physiol 1999 276 L858 L867 10.1152/ajplung.1999.276.5.L858 10330042

[B55] Been JV Debeer A van Iwaarden JF Kloosterboer N Passos VL Naulaers G Zimmermann LJ Early alterations of growth factor patterns in bronchopulmonary dysplasia Pediatr Res 2010 67 83 89 10.1203/PDR.0b013e3181c13276 19770691

[B56] Thickett DR Armstrong L Christie SJ Milar AB Vascular endothelial growth factor may contribute to increased vascular permeability in acute respiratory distress syndrome Am J Respir Crit Care Med 2001 164 1601 1605 11719296 10.1164/ajrccm.164.9.2011071

[B57] Thickett DR Armstrong L Milar AB A role for vascular endothelial growth factor in acute and resolving lung injury Am J Respir Crit Care Med 2002 166 1332 1337 10.1164/rccm.2105057 12421742

[B58] Carne J Chupp G Lee CG Homer RJ Zhu Z Chen Q Ma B Du Y Roux F McArdle J Waxman AB Elias JA IL-13 stimulates vascular endothelial cell growth factor and protects against hyperoxic acute lung injury J Clin Invest 2000 106 783 791 10.1172/JCI9674 10995789 PMC381393

[B59] Pryhuber G O'Brien D Baggs R Phipps R Huyck H Sanz I Nahm M Ablation of tumor necrosis factor receptor type I (p55) alters oxygen-induced lung injury Am J Physiol Lung Cell Mol Physiol 2000 278 L1082 1090 10781441 10.1152/ajplung.2000.278.5.L1082

[B60] Guthmann F Wissel H Schachtrup C Tölle A Rüdiger M Spener F Rüstow B Inhibition of TNFalpha in vivo prevents hyperoxia-mediated activation of caspase 3 in type II cells Resp Res 2005 6 10 25 10.1186/1465-9921-6-10 PMC548140 15663790

[B61] Wendel M Giessmann U Behrend P Augstein A Koslowski R Haufe D Kasper M Koch T Inflammatory-activated microvascular endothelial cells regulate interleukin-8 and monocyte chemoattractant protein-1 expression of A549 cells in a paracrine fashion Exp Lung Res 2008 34 85 100 10.1080/01902140701807910 18266131

[B62] Zhu Y Miller TL Singhaus CJ Shaffer TH Chidekel A Effects of oxygen concentration and exposure time on cultured human airway epithelial cells Pediatr Crit Care Med 2008 9 224 229 10.1097/PCC.0b013e318166fbb5 18477937

[B63] Zhong J Deaciuc IV Burikhanov R de Villers WJ Lipopolysaccharide-induced liver apoptosis is increased in interleukin-10 knockout mice Biochemi Biophys Acta 2006 1762 468 477 10.1016/j.bbadis.2005.12.012 16497487

[B64] Thannickal VJ Fanburg BL Reactive oxygen species in cell signaling Am J Physiol Lung Cell Mol Physiol 2000 279 1005 1028 10.1152/ajplung.2000.279.6.L1005 11076791

[B65] McAuliffe PF Murday ME Efron PA Scumpia PO Ungaro R Abouhamze A Tannahill CL Hutchins B Laface D Moldawer LL Dose-dependent improvements in outcome with adenoviral expression of interleukin-10 in a murine model of multisystem organ failure Gene Ther 2006 13 276 282 10.1038/sj.gt.3302600 16251998

